# A Novel Mutation in the Upstream Open Reading Frame of the *CDKN1B* Gene Causes a MEN4 Phenotype

**DOI:** 10.1371/journal.pgen.1003350

**Published:** 2013-03-21

**Authors:** Gianluca Occhi, Daniela Regazzo, Giampaolo Trivellin, Francesca Boaretto, Denis Ciato, Sara Bobisse, Sergio Ferasin, Filomena Cetani, Elena Pardi, Márta Korbonits, Natalia S. Pellegata, Viktoryia Sidarovich, Alessandro Quattrone, Giuseppe Opocher, Franco Mantero, Carla Scaroni

**Affiliations:** 1 Department of Medicine, Endocrinology Unit, University of Padova, Padova, Italy; 2 Department of Endocrinology, Barts and the London School of Medicine, Queen Mary University of London, London, United Kingdom; 3 Familial Cancer Clinic and Oncoendocrinology, Veneto Institute of Oncology, IRCCS, Padova, Italy; 4 Department of Endocrinology and Metabolism, Section of Endocrinology and Bone Metabolism, University of Pisa, Pisa, Italy; 5 Institute of Pathology, Helmholtz Zentrum München, German Research Center for Environmental Health, Neuherberg, Germany; 6 Laboratory of Translational Genomics, Centre for Integrative Biology, University of Trento, Trento, Italy; 7 Department of Medicine, University of Padova, Padova, Italy; University of Washington, United States of America

## Abstract

The *CDKN1B* gene encodes the cyclin-dependent kinase inhibitor p27^KIP1^, an atypical tumor suppressor playing a key role in cell cycle regulation, cell proliferation, and differentiation. Impaired p27^KIP1^ expression and/or localization are often observed in tumor cells, further confirming its central role in regulating the cell cycle. Recently, germline mutations in *CDKN1B* have been associated with the inherited multiple endocrine neoplasia syndrome type 4, an autosomal dominant syndrome characterized by varying combinations of tumors affecting at least two endocrine organs. In this study we identified a 4-bp deletion in a highly conserved regulatory upstream ORF (uORF) in the 5′UTR of the *CDKN1B* gene in a patient with a pituitary adenoma and a well-differentiated pancreatic neoplasm. This deletion causes the shift of the uORF termination codon with the consequent lengthening of the uORF–encoded peptide and the drastic shortening of the intercistronic space. Our data on the immunohistochemical analysis of the patient's pancreatic lesion, functional studies based on dual-luciferase assays, site-directed mutagenesis, and on polysome profiling show a negative influence of this deletion on the translation reinitiation at the *CDKN1B* starting site, with a consequent reduction in p27^KIP1^ expression. Our findings demonstrate that, in addition to the previously described mechanisms leading to reduced p27^KIP1^ activity, such as degradation via the ubiquitin/proteasome pathway or non-covalent sequestration, p27^KIP1^ activity can also be modulated by an uORF and mutations affecting uORF could change p27^KIP1^ expression. This study adds the *CDKN1B* gene to the short list of genes for which mutations that either create, delete, or severely modify their regulatory uORFs have been associated with human diseases.

## Author Summary

Gene expression can be modulated at different steps on the way from DNA to protein including control of transcription, translation, and post-translational modifications. An abnormality in the regulation of mRNA and protein expression is a hallmark of many human diseases, including cancer. In some eukaryotic genes translation can be influenced by small DNA sequences termed upstream open reading frames (uORFs). These elements located upstream to the gene start codon may either negatively influence the ability of the translational machinery to reinitiate translation of the main protein or, much less frequently, stimulate protein translation by enabling the ribosomes to bypass *cis*-acting inhibitory elements. *CDKN1B*, which encodes the cell cycle inhibitor p27^KIP1^, includes an uORF in its 5′UTR sequence. p27^KIP1^ expression is often reduced in cancer, and germline mutations have been identified in *CDKN1B* in patients affected with a syndrome (MEN4) characterized by varying combinations of tumors in endocrine glands. Here we show that a small deletion in the uORF upstream to *CDKN1B* reduces translation reinitiation efficiency, leading to underexpression of p27^KIP1^ and coinciding with tumorigenesis. This study describes a novel mechanism by which p27^KIP1^ could be underexpressed in human tumors. In addition, our data provide a new insight to the unique pathogenic potential of uORFs in human diseases.

## Introduction

*CDKN1B* encodes the cyclin-dependent kinase (CDK) inhibitor, p27^KIP1^, which negatively regulates the Cdk2/cyclin E and Cdk2/cyclin A protein complexes, thereby preventing the progression from the G1 to the S phase of the cell cycle [1]. In G0 and early G1, p27^KIP1^ expression and stability are maximal. During the G1 phase gradual degradation of p27^KIP1^ is associated with an increased activity of Cdk2/cyclin E and Cdk2/cyclin A complexes to stimulate cell proliferation [2], [3]. Several mitogenic (i.e. MAPK, PI3K/AKT) and anti-proliferative (i.e. TGFβ/SMAD) signal transduction pathways regulate p27^KIP1^ expression and activity, making it a central integration point for cell-fate decision [4]. These pathways can regulate p27^KIP1^ at different levels, including transcription, translation, intracellular localization or ubiquitin-mediated proteasomal degradation [5].

p27^KIP1^ acts as an atypical tumor suppressor as it is rarely mutated in human cancers, but frequently underexpressed or mislocalized in human malignancies [4]. Although an augmented proteolysis was initially suggested as the major cause of p27^KIP1^ loss in human tumors [6], recent findings propose that reduced translation and/or transcription of *CDKN1B* also contributes to p27^KIP1^ deficiency [7]–[9].

Translation of *CDKN1B* may involve regulatory elements within its 5′UTR, including an internal ribosome entry site (IRES) and an upstream ORF (uORF) [10], [11]. The IRES supports p27^KIP1^ expression when cap-dependent translation is reduced, such as during quiescence or stress conditions [10], [12]. Reduced IRES-mediated translation, due to mutations in the pseudouridine synthase that alters the ribosome's ability to efficiently engage the *CDKN1B* IRES element, may contribute to the increased predisposition to cancer in X-linked congenital dyskeratosis [13].

Germline mutations in the *CDKN1B* gene have been recently associated with the development of a multiple endocrine neoplasia syndrome both in humans (MEN4, MIM 610755) and in rats (MENX) [14]. Multiple endocrine neoplasias, including type 1 (MEN1, MIM 131100) and type 2 variants, (MEN2, MIM 171400, MIM 162300), are a group of autosomal dominant syndromes characterized by varying combinations of tumors affecting at least two endocrine organs [15].

To date, seven *CDKN1B* germline mutations have been identified in MEN4 patients primarily associated with MEN1-related lesions, including parathyroid and pituitary tumors, but the presence of other malignancies such as renal angiomyolipoma, papillary thyroid carcinoma and pancreatic masses has also been reported [8], [9], [14], [16], [17]. Two further germline mutations have been more recently associated with sporadic hyperparathyroidism [18]. In MEN4, *CDKN1B* mutations either affect p27^KIP1^ cellular localization, protein stability or the binding with functional partners such as Cdk2 or Grb2 [8], [17]. Reduced transcription/translation efficiency due to mutations in elements regulating translation initiation (i.e., in the Kozak sequence, or forming a secondary stem loop structure within the *CDKN1B* 5′UTR), has also been described [8], [9].

Germline *CDKN1B* mutations are hence rare events in MEN1-like subjects (individuals with MEN1-related lesions, without *MEN1* inactivating mutations), being identified in less than 3% of cases [8], [16] and a clear genotype-phenotype correlation has not been established to date.

In the present paper we analyzed the *CDKN1B* gene looking for point mutations and large rearrangements in order to determine the possible cause of multiple endocrine tumors in 25 consecutive sporadic and familial patients with typical MEN1-related symptoms. We identified a 4-bp deletion that modifies the regulatory uORF in the 5′UTR of the *CDKN1B* gene in a patient with tumors in the pituitary gland and the endocrine pancreas. Functional studies based on dual-luciferase assay and site-directed mutagenesis further support the deleterious influence of this deletion on translation reinitiation at the *CDKN1B* starting site, with a consequent reduction of p27^KIP1^ expression both *in vitro* and *in vivo*.

## Results/Discussion

### The regulatory uORF within the *CDKN1B* gene is mutated in an acromegalic patient affected by a well-differentiated pancreatic lesion

Among the 25 patients with MEN1-related symptoms, a 4-bp deletion (c.-456_-453delCCTT, NM_004064) within the 5′UTR of *CDKN1B* in a 62 year old female patient with acromegaly and a well-differentiated non-functioning pancreatic endocrine neoplasm has been identified. This sequence variant was not detected in either 600 chromosomes or in the dbSNP/1000 genomes databases.

**Figure 1:**
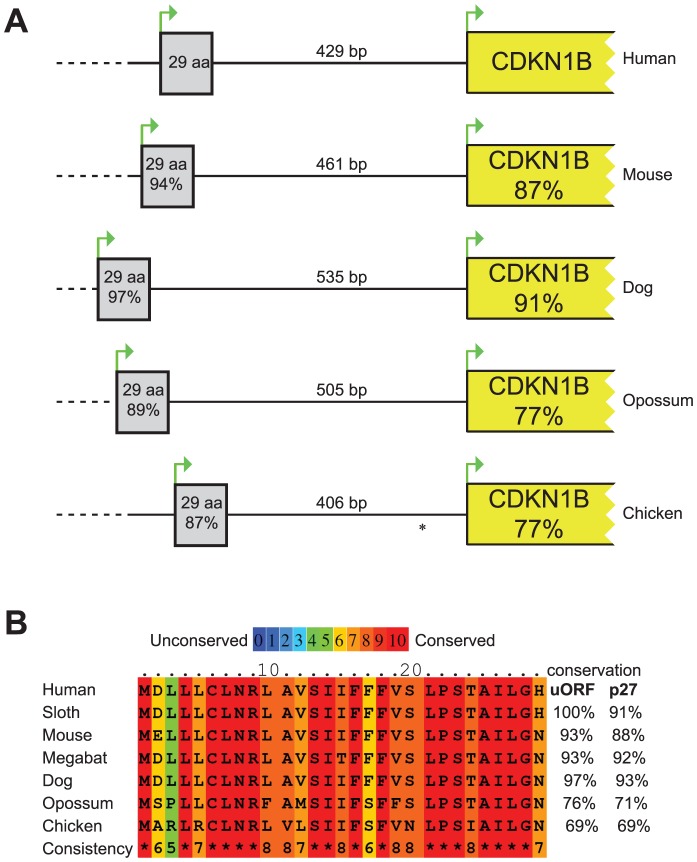
Comparison of the *CDKN1B* gene among species. (A) Schematic representation of the *CDKN1B* gene in different species. The uORF has been reported as grey box while *CDKN1B* as a yellow one. Similarities at nucleotide level with human sequences have been reported within boxes. Intercistronic length is also shown. * In chicken *CDKN1B* 5′UTR a second uORF has been described. (B) uORF amino acid sequences in seven species. In all but one, conservation of the uORF encoded peptide is even higher than p27^KIP1^. The considered nucleotide and protein sequences are the following: Human, NM_004064.3, NP_04055.1; Mouse, NM_009875.4, NP_034005.2; Dog, HE804769, CCH35981; Opossum, HE804772, CCH35984; Chicken, NM_204256, NP_989587; Sloth, HE804770, CCH35982; Megabat, HE804771, CCH35983. Nucleotide and protein sequences alignments among species have been performed by LALIGN and PRALINE, respectively.

The 5′UTR of the *CDKN1B* gene is highly structured, containing several translational regulatory elements. An IRES element sustains p27^KIP1^ translation under poor growth conditions [10], [12], while a G/C-rich hairpin domain contributes to cell-cycle dependent regulation of *CDKN1B* translation [11]. Downstream the G/C-rich domain and encompassing the c.-456_-453delCCTT, an uORF coding for a 29 amino acid-long peptide has been described that has been suggested to inhibit the *in vitro* synthesis of p27^KIP1^ and to enhance its cell cycle-dependent translation [11]. An extensive comparative analysis of DNA and protein sequences from multiple species (Figure 1) confirmed previous data of high evolutionary conservation among vertebrates of the uORF [11], and support the hypothesis of a functional role of this element [11].

In general, uORFs are small open reading frames located in the 5′UTR of genes that influence translation during ribosome scanning, thus modulating gene expression. A scanning ribosome encountering an uORF has multiple fates: it can i) translate the uORF; ii) scan through the sequence (leaky scanning) and reinitiate translation further downstream at a proximal or distal ATG; iii) induce ribosome stalling or premature dissociation at the uORF stop codon, thus reducing downstream-cistron translation [19] or down-regulating gene expression by promoting mRNA decay [20].

**Figure 2:**
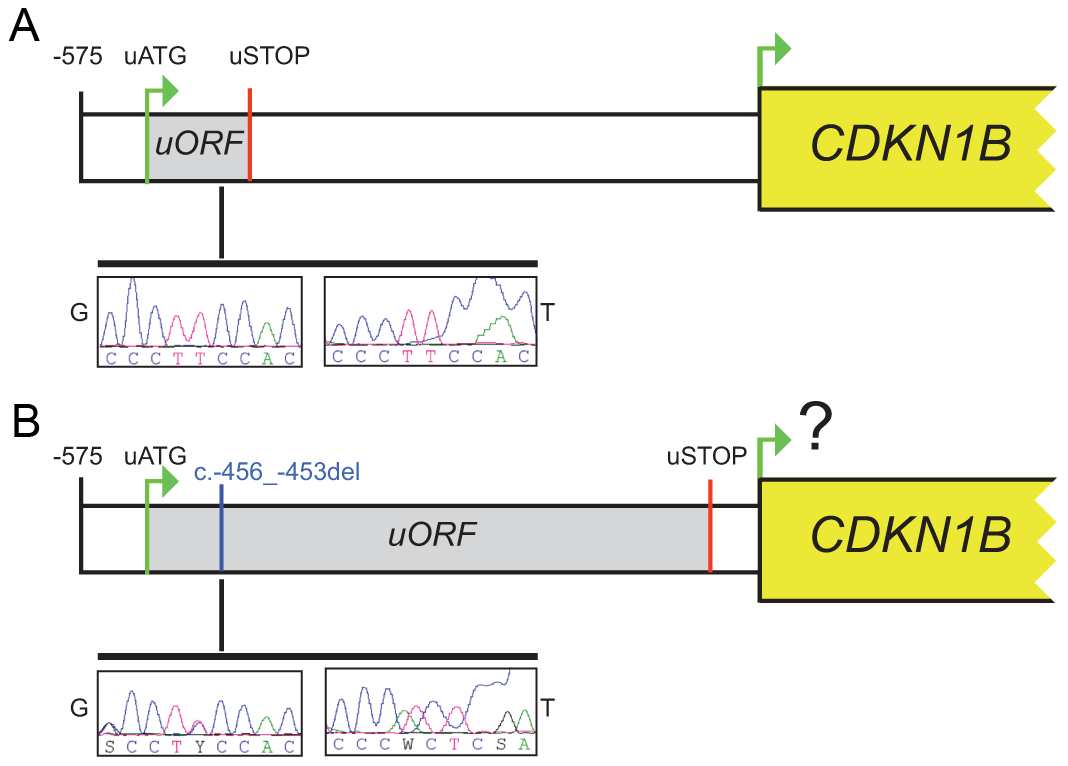
The c.-456_-453delCCTT affects protein translation by reducing reinitiation. Schematic representation of the *CDKN1B* 5′UTR in the wild type (A) and in the c.-456_-453delCCTT carrier (B). The uORF coding sequence is shown as grey box. Electropherograms for germline (G) and tumor (T) DNA are shown.

In our case the 4-bp deletion shifts the uORF termination codon, thus lengthening the uORF encoded peptide from 29 to 158 amino acids and shortening the intercistronic space from 429 to 38 bp, with a possible negative influence on translation reinitiation from the main ATG (Figure 2). Long uORFs and short intercistronic regions may indeed prevent the 40S ribosomal subunits from keeping and/or re-acquiring appropriate cofactors for translation resumption/reinitiation at the downstream ATG [21], [22].

### The c.-456_-453delCCTT affects p27^KIP1^ expression without altering neither the steady-state level of *CDKN1B* mRNA nor the promoter usage

**Figure 3:**
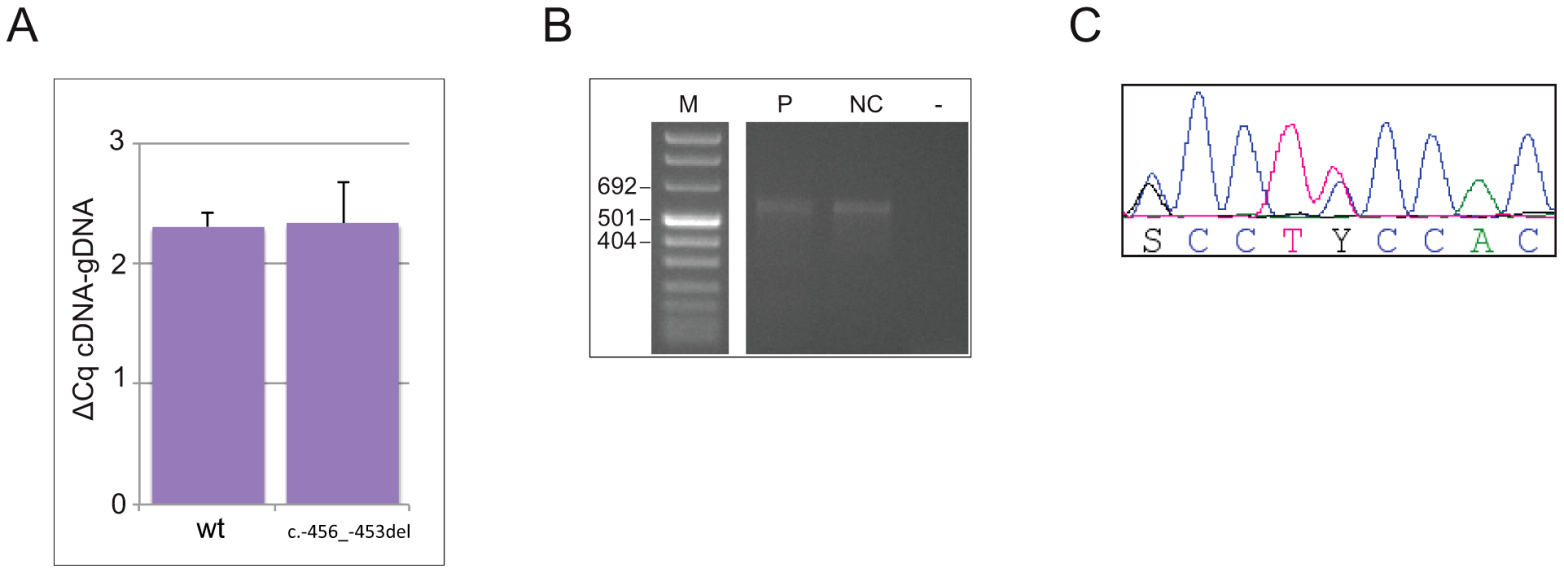
The c.-456_-453delCCTT does not alter the steady-state levels of *CDKN1B* allelic mRNA nor the promoter usage pattern. (A) The wild type and mutated alleles were present at similar level in blood-derived RNA. The amount of both alleles are expressed as differences between Cq values obtained for mRNA minus Cq for genomic DNA for removing the intrinsic variation between the two qPCR assays. (B) A single *CDKN1B* promoter is used in lymphocytes from both deletion carrier (P) and a normal control (NC). M, molecular marker; -, negative control. (C) Both wild type and mutated alleles are transcribed in pancreatic lesion.

To address the possibility that the 4-bp deletion affects transcription and/or mRNA stability, making a decreased translation rate due to reduced reinitiation efficiency biologically irrelevant, or alters the promoter usage pattern preventing transcription of the uORF-containing isoform [10], we measured the steady state levels of *CDKN1B* allelic mRNAs from whole blood by 5′RACE and allele-specific qPCR. As reported in Figure 3a, both wild type and mutated alleles were expressed in blood cells in almost equal amounts, suggesting that the identified deletion does not alter mRNA steady state levels, and therefore probably does not alter either transcription or mRNA stability. An apparently unique 5′UTR of >530 bp has been identified in the c.-456_-453delCCTT carrier and in healthy controls, supporting the concept that the transcription pattern is preserved in the mutated subject (Figure 3b).

**Figure 4:**
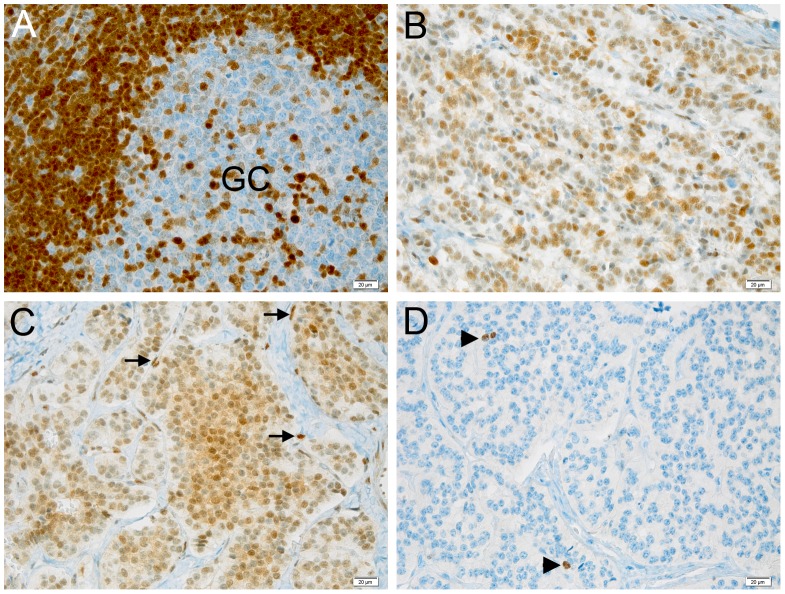
Expression of p27^KIP1^ and Ki-67 in pancreatic tumors and control tissue. (A–C) Immunohistochemical staining with anti-p27^KIP1^ antibody. (D) Staining with anti-Ki67 antibody. (A) staining of a control human tonsil. Cells in the lymphatic nodule show the expected strong nuclear positivity for p27^KIP1^, while only few of the highly proliferating germinal center (GC) cells express p27^KIP1^. (B) Sporadic endocrine pancreatic tumor of an individual with no known germline mutations. The tumor cells show intermediate levels of nuclear immunoreactivity for p27^KIP1^. (C) Endocrine pancreatic tumor of the patient having the c.-456_-453delCCTT mutation. Tumor cells show low expression of p27^KIP1^ in the nucleus but also expression in the cytoplasm. Endothelial cells (arrows) show the typical strong nuclear positivity and serve as internal control to check for the adequacy of the staining. (D) Same tissue as in C stained for the proliferation marker Ki-67. Positive nuclei are indicated with arrowheads. The proliferation rate was estimated to be ca. 1%. Bar, 20 µM. Original magnification: A–D, 200×.

The pancreatic tumor of the mutated patient was then analyzed by immunohistochemistry for p27^KIP1^ expression and for the proliferation antigen Ki-67, and compared with similar tumors from *CDKN1B*-mutation negative subjects. Parallel differences in expression level and localization were found. We observed weak cytoplasmic staining in tumor cells and very strong nuclear staining in the interspersed normal endothelial cells in the MEN4 patient (Ki67<1%), while in contrast p27^KIP1^ nuclear staining was found in a high proportion of sporadic well-differentiated pancreatic tumors examined (Figure 4). The reduction in nuclear p27^KIP1^ and/or its cytoplasmic mislocalization has been reported in different cancers including breast, colon and prostate [4]. Loss of p27^KIP1^ may occur through different mechanisms, including augmented proteasome-mediated proteolysis and impaired translation [23]. On the other hand, the cytoplasmic mislocalization may be associated with imbalanced p27^KIP1^ phosphorylation due to the oncogenic activation of PI3K- and MEK-dependent kinases, mimicking protein loss [4]. Indeed, in the cytoplasm p27^KIP1^ is unable to exert its inhibitory activity on CDK even in the presence of anti-mitogenic stimuli.

On the same lesion loss of heterozygosity (LOH) analysis was then performed. No loss of the wild type allele was observed (Figure 2). Moreover, the biallelic expression of an uORF-containing transcript has been observed (Figure 3c), further confirming that p27^KIP1^ may act as a haploinsufficient tumor suppressor [24].

### The c.-456_-453delCCTT affects p27^KIP1^ translation reinitiation by altering *CDKN1B* uORF length and the intercistronic distance in a cell cycle–independent manner

To identify possible additional uORF mutations, we extended the *CDKN1B* 5′UTR analysis to additional 41 patients with typical MEN1-like features previously reported negative for mutations in the *CDKN1B* coding sequence [17], [25]. A c.-469C>T substitution resulting in a silent change in the uORF was detected in a single patient but not in healthy controls (see above).

**Figure 5:**
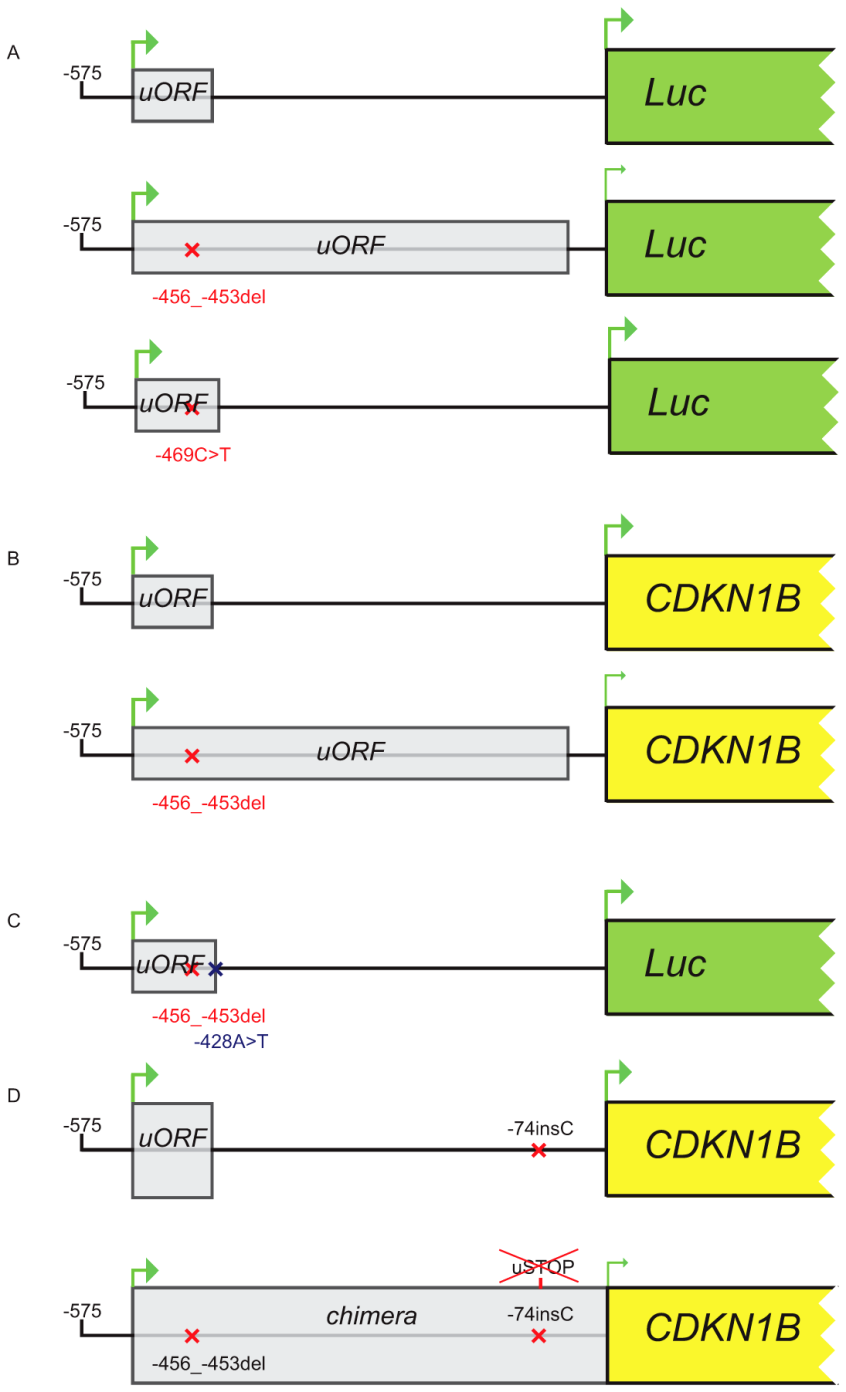
Constructs used in transfection experiments. (A) Either the wild type, the c.-456_-453delCCTT or the c.-469C>T containing 5′UTRs were cloned upstream the firefly luciferase gene. (B) The wild type/c.-456_-453delCCTT 5′UTR were subcloned upstream the *CDKN1B* gene. (C) The introduction of the c.-428A>T substitution in the two former plasmids reported in panel A restores uORF length and intercistonic distance. (D) Constructs reported in panel B were mutated introducing a c.-74insC leading to the translation of a chimeric protein in the double mutant.

**Figure 6:**
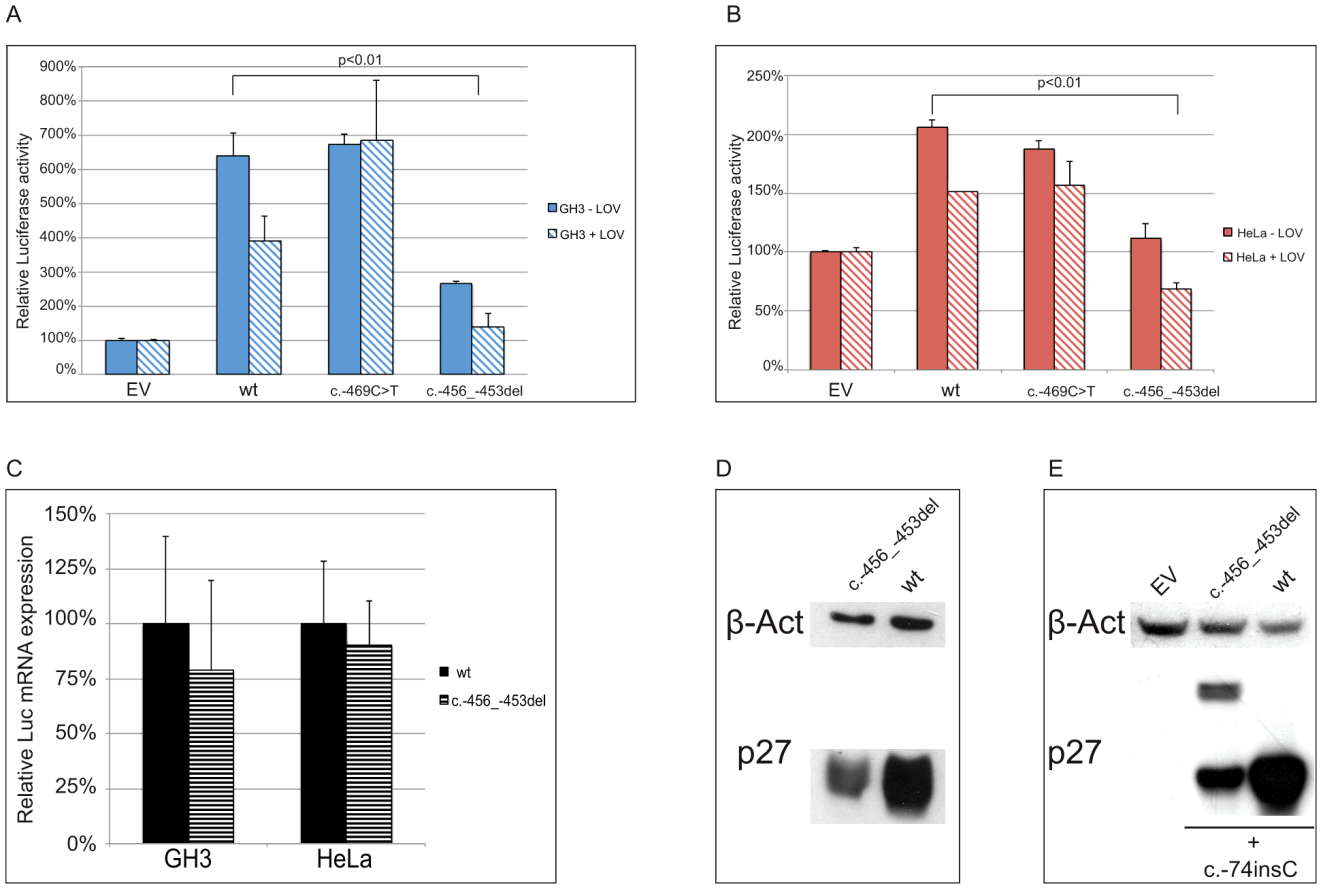
The c.-456_-453delCCTT affects protein translation by reducing reinitiation. Relative luciferase activity for the wild type and the mutants (c.-456_-453delCCTT, c.-469C>T) 5′UTRs was measured in GH3 (A) and HeLa (B) cells. The effects of the 4 bp deletion on luciferase mRNA in HeLa and GH3 cells (C) and on p27^KIP1^ level in HEK293 cells (D) are shown. Panel E shows the presence of the chimeric product in HEK293 cells transfected with the double mutant construct (c.-456_-459delCCTT+c.-74insC), followed by substantial reduction of p27^KIP1^ expression, demonstrated by western blot. P-values were calculated using a two-tailed t-test. *p<0,01; EV, empty vector; wt, wild type; error bars, standard deviation.

To determine whether the two identified substitutions negatively affect *CDKN1B* translation, the wild type and mutated 5′UTRs were cloned upstream of the firefly luciferase gene (Figure 5a). By transfecting lovastatin G1-synchronized or asynchronous HeLa and GH3 cells, we demonstrated that the c.-456_-453delCCTT, but not the c.-469C>T variant, significantly reduced luciferase activity in a cell cycle phase-independent manner (Figure 6a, 6b). When we analyzed the luciferase mRNA from the transfected cells by quantitative real-time RT-PCR, we demonstrated that the effects of the 4-bp deletion are largely due to reduction in translation rate rather than to changed steady-state mRNA levels (Figure 6c), in agreement with our observation on blood *CDKN1B* mRNA (Figure 3a) and with the trend observed in large-scale datasets [26].

We then evaluated the effect of the 4 bp deletion on p27^KIP1^ translation by transfecting HEK293 cells with vectors with either the wild type or the mutated 5′UTRs cloned upstream the *CDKN1B* gene (Figure 5b). We confirmed a significant reduction in p27^KIP1^ protein levels as a consequence of the 5′UTR c.-456_-453delCCTT mutation (Figure 6d).

In a previous study on HeLa cells using an identical wild type construct, the *CDKN1B* 5′UTR induced luciferase expression only during G1 progression or in lovastatin-arrested cells [11]. Although we cannot exclude the presence of DNA variations on regulatory elements between the two cloned sequences, a possible biological variability between batches of cells from the same cell line seems the more plausible explanation. However, similar cell-cycle independent luciferase activation was observed under our experimental conditions in three additional cell lines, namely GH3 (Figure 6a), SH-SY5Y and HEK293 (Figure S1). Based on such observation we may therefore suggest the need for further studies for better clarifying the cell-cycle dependent translation of p27^KIP1^ regulated by the *CDKN1B* 5′UTR.

**Figure 7:**
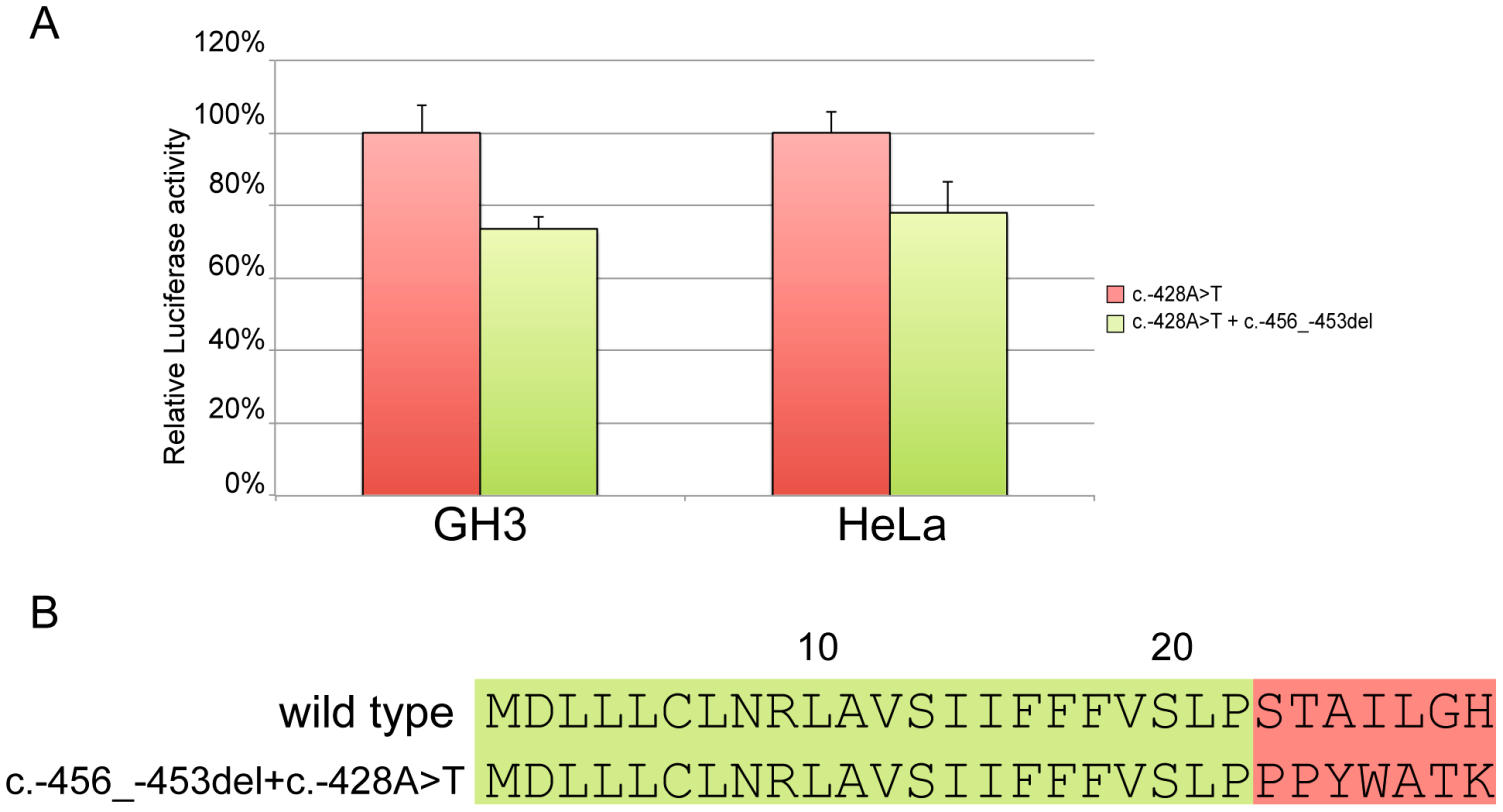
Effect of the c.-428A>T restoring mutation. In both GH3 and HeLa cell lines c.-428A>T almost restores the modulation properties of the *CDKN1B* wild type uORF (A). Panel B shows the primary sequence differences in the wild type uORF compared with the -456_-453delCCTT+c.-428A>T.

Site-directed mutagenesis (c.-428A>T) was then used to reintroduce a stop codon in the c.-456_-453delCCTT containing vector, thus restoring both uORF length and intercistronic distance (Figure 5c). After transfection, the uORF regulatory properties were almost completely rescued in the double mutant compared to the c.-428A>T construct (Figure 7a), further supporting the hypothesis that the 4 bp deletion affects translation reinitiation of the downstream *CDKN1B* ORF. In addition, the lack of complete recovery of the uORF modulatory activity, possibly due to differences in the C-terminus of the uORF-encoded peptide (Figure 7b), further confirms that the *CDKN1B* uORF belongs to the class of sequence-dependent uORFs that exert their inhibitory role by acting in *cis* to regulate components of the translation apparatus [19].

### The *CDKN1B* uORF is efficiently translated

To evaluate the ability of the uORF to be translated, which represents the central point of our hypothesis on the possible deleterious effects of the c.-456_-453delCCTT change, the wild type or the mutated 5′UTRs were placed upstream of the *CDKN1B* open reading frame and the c.-74insC mutation was introduced by site-directed mutagenesis. This additional DNA variant leads to the in-frame fusion of the mutated uORF with the main gene (Figure 5d). As expected, the chimeric product was detected only in the c.-74insC+c.-456_-453delCCTT transfected HEK293 cells, and was again associated with a significant reduction of p27^KIP1^ expression (Figure 6e). To our knowledge, this is the first direct evidence of the translation of the *CDKN1B* uORF in a cellular system. However, it remains to be clarified if this peptide has additional biological functions other than repressing translation of the *CDKN1B* ORF as an effect of impaired reinitiation, as suggested for a subset of uORFs [27].

### The c.-456_-453delCCTT transcript is less efficiently loaded onto polysomes than the wild-type one

**Figure 8:**
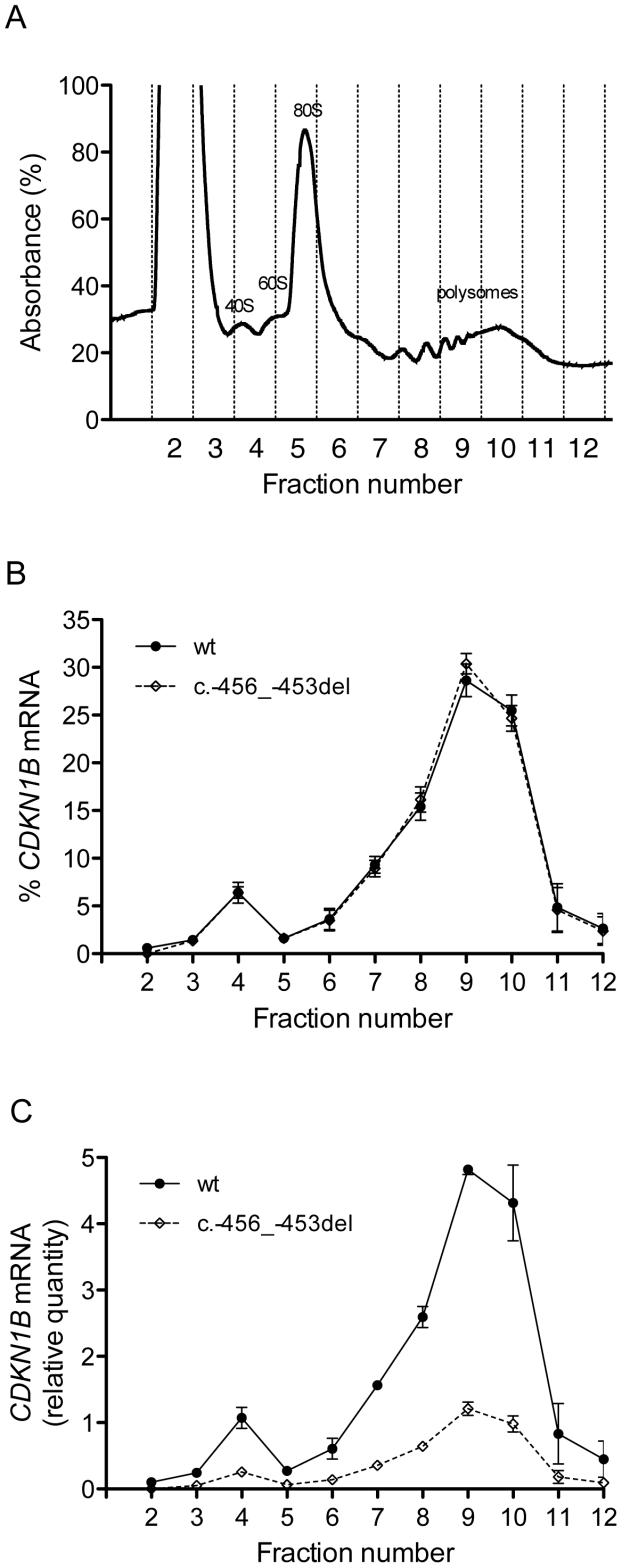
Polysome profiling of lymphoblastoid cells from the -456_-453delCCTT mutation carrier. (A) Representative absorbance profile for RNA separated by velocity sedimentation through a 15–50% sucrose gradient. The positions of the 40S, 60S, 80S, and polysomal peaks are indicated. (B) The levels of *CDKN1B* mRNA (wt and c.-456_-453del) in each gradient fraction were measured by quantitative real-time PCR and plotted as a percentage of the total *CDKN1B* mRNA levels (wt or c.-456_-453del, respectively) in that sample. Data represent three independent experiments, with mean ± SD reported. (C) The levels of both alleles in each fraction were expressed as relative quantities calculated from differences in Cq values between mRNA and genomic DNA for removing the intrinsic variation between the two qPCR assays. Data represent the mean of three independent experiments ± SD.

To elucidate the molecular mechanism by which c.-456_-453delCCTT determines a decrease in p27^KIP1^ translational efficiency, we estimated the relative proportion of the two allelic mRNAs engaged in translation in the immortalized lymphoblastoid cells of the heterozygote patient. To this aim, the cell lysates were subjected to polysome fractionation through sucrose gradient ultracentrifugation [28] and we determined the level of each of the two allelic mRNAs for each fraction. Figure 8a reports the distribution of ribosomal RNA in the different fractions, showing a typical distribution with polysomes reproducibly spanning fractions 7–11. The distributions of the wild type and c.-456_-453delCCTT transcripts present an almost superimposable pattern, being for both about 90% of the total detectable mRNA localized in polysomes with a peak corresponding to fraction 9 (compare Figure 8b with Figure 8a). However, when the amounts of both alleles were expressed as differences between Cq values for mRNA and for genomic DNA for removing the intrinsic variation between the two qPCR assays, a clear preponderance on polysomes of the wild type *CDKN1B* mRNA could be observed (Figure 8c), which we can estimate to be of the order of about three times. Since the levels of the two allelic mRNAs in the cells are the same (Figure 3a), this implies that the c.-456_-453delCCTT mRNA suffers decreased average polysomal loading with respect to the wild type mRNA. Therefore, the two different *CDKN1B* mRNAs are differentially loaded in polysomes despite being present in the cells in the same relative amounts, and despite the fact that they share a distribution profile on polysomes of different molecular weights. The result is compatible with a decreased efficiency of translation reinitiation of the *CDKN1B* ORF due to the c.-456_-453delCCTT mutation.

### The role of uORF mutations in MEN4 and other human diseases

The data we presented here further confirm the role of *CDKN1B* germline mutations in predisposing to a MEN4 syndrome. Furthermore, they demonstrate that a reduced translation initiation rate of p27^KIP1^ due to the ineffective regulatory activity of its uORF may be associated with transformation. Based on our data, mutations in the *CDKN1B*-regulating uORF seem to be rare. However, previous studies on *CDKN1B* germline mutations in MEN1-like patients did not consider the uORF region [8], [9], [14], [16]–[18], [25], [29], and therefore the prevalence of this type of mutation remains to be established. Our results emphasize thus the need for the inclusion of the entire 5′UTR region of *CDKN1B* in molecular testing for MEN4.

Increasing evidence suggests that uORF-mediated translational control may represent an important mechanism in the regulation of gene expression. This is supported by the close relationship of mutations that introduce or disrupt uORFs and the pathophysiology of several human diseases, including cancer [30]. To date, only three well-known hereditary diseases have been associated with uORF-affecting mutations: i) thrombocythemia due to thrombopoietin mutation [31], ii) melanoma due to *CDKN2A* mutation [32] and iii) Marie Unna hypotrichosis due to mutations in the *hairless* gene [33]. Other diseases, such as breast cancer, Alzheimer's diseases, arrhythmogenic right ventricular cardiomyopathy have also been suggested to be associated to genes which have uORF-related control [34]–[36]. However, the pathogenic effects of deregulated uORF-mediated translation in these cases remain to be clarified [37].

Many important genes involved in controlling cell growth (i.e. receptors, oncogenes, growth factors) harbor uORF in their 5′UTR [38]. Some of these genes override the uORF-mediated translational repression and accumulate their protein product in cancer cells [39]. Translational derepression elements in the 3′UTR may counteract the inhibitory activity of uORFs on translation [39]; however, mutations inducing loss of uORF function in oncogenes might lead to a similar increase of translation rate and consequently to malignant transformation. Conversely, gain of function mutations in uORFs regulating tumor suppressor genes may reduce translation of protective proteins leading to tumor formation [32]. Similarly to a point mutation introducing a regulative uORF in the leader sequence of the tumor suppressor gene *CDKN2A* in hereditary melanoma [32], the 4-bp deletion in *CDKN1B* gene we describe here led to the reduced production of *CDKN1B*-encoded protein p27^KIP1^, probably due to a decreased translation reinitiation rate, which then results in predisposition to tumor development.

### Conclusions

In conclusion, the *CDKN1B* mutation functionally characterized in this study represents a novel example of an uORF-affecting mutation. Our functional studies show the negative influence of this deletion on the translation reinitiation at the *CDKN1B* starting site thus providing novel insights into the role of uORFs in the pathogenesis of human diseases.

In addition to the classical mechanisms of degradation by the ubiquitin/proteasome pathway and by non-covalent cytoplasmic sequestration, our findings demonstrate that p27^KIP1^ activity can also be modulated by its uORF, and mutations affecting this sequence may lead to reduced expression of p27^KIP1^ protein.

## Materials and Methods

### Patients

The cohort of patients screened for mutations in the entire *CDKN1B* gene consisted of 25 consecutive patients with two or more typical MEN1-related symptoms (hyperparathyroidism, neuroendocrine tumors, pituitary adenoma). Patients were collected and diagnosed at the Division of Endocrinology (University/Hospital of Padova) and at the Familial Cancer Clinic and Oncoendocrinology (Veneto Institute of Oncology), Padova, Italy, following the recognized clinical practice guidelines [40]. All patients had negative mutational screening for *MEN1*, *PRKAR1A* and *AIP* genes. A second group of additional 41 patients with similar phenotype has been analyzed only for the uORF sequence since the rest of the gene has been analyzed and published previously without finding any pathogenic mutations [17], [25]. The study was conducted in accordance with the Helsinki declaration. Local ethical committees from each referring center approved the study, and all subjects gave written informed consent.

### Mutational analysis for *CDKN1B* gene and LOH

The whole coding region, intron–exon boundaries, and 5′- and 3′-UTRs of *CDKN1B* were amplified and directly sequenced as reported elsewhere [41]. All primer pairs used were designed by PRIMER3 (http://primer3.sourceforge.net/) and synthesized by IDT (Leuven, Belgium). Primers for point mutation analysis of the entire human *CDKN1B* gene were P0F, 5′-agcagtacccctccagcagt-3′; P0R, 5′-aaagcccgtccgagtctg-3′; P1F, 5′-ccaatggatctcctcctctg-3′; P1R, 5′-ggagccaaaagacacagacc-3′; P2F, 5′-ccatttgatcagcggagact-3′; P2R, 5′-gccctctaggggtttgtgat-3′; P3F, 5′-gagttaacccgggacttggag-3′; P3R, 5′-atacgccgaaaagcaagcta-3′; P4F, 5′-tgactatggggccaacttct-3′; P4R, 5′-tttgccagcaaccagtaaga-3′; P5F, 5′-ccccatcaagtatttccaagc-3′; P5R, 5′-cctcccttccccaaagttta-3′; P6F, 5′-tgcctctaaaagcgttggat-3′; P6R, 5′-tttttgccccaaactacctg-3′; P7F, 5′-gccctccccagtctctctta-3′; P7R, 5′-ggtttttccatacacaggcaat-3′; P8F, 5′-tctgtccatttatccacaggaa-3′; P8R 5′-tgccaggtcaaataccttgtt-3′.

Previously unreported nucleotide changes were screened in 300 healthy, anonymous, unrelated individuals by Tetra-primer ARMS-PCR [42] and searched in the dbSNP and 1000 genomes databases (http://www.ncbi.nlm.nih.gov/projects/SNP/; http://www.1000genomes.org/). The NHLBI Exome Sequencing Project - Exome Variant Server database (http://evs.gs.washington.edu/EVS) has been queried for the c.-469C>T. Primers for Tetra-primer ARMS-PCR were: hp27delOUTR, 5′-agccgctctccaaacctt-3′; hp27delOUTF, 5′-caatggatctcctcctctgttt-3′; hp27delINF, 5′-cttcttcgtcagcctcccac-3′; hp27-469INR, 5′-tggcggtggaagggaggctgacgcaa-3′; hp27-469INF, 5′-gactcgccgtgtcaatcattttcgtc-3′.

Gene dosage alteration was assessed by the quantitative multiplex PCR of short fluorescent fragments (QMPSFs) and by long-range PCR (LR-PCR) as previously described [41] using the following primers: CLIF, 5′-tggtcagagagtggcctttctc-3′; CLIR, 5′-tgccgagtagaggcatttagtca-3′; CLIIF, 5′-tgtctgtgacgccgttgtct-3′; CLIIR, 5′-aagggttttctagcacacataggaa-3′; 1IF, 5′-gccgcaaccaatggatctc-3′; 1IR, 5′-acgagccccctttttttagtg-3′; 1IIF, 5′-ctctgaggacacgcatttggt-3′; 1IIR, 5′-aaatcagaatacgccgaaaagc-3′; 2F, 5′-tttcccctgcgcttagattc-3′; 2R, 5′-ccaccgagctgtttacgtttg-3′; 3IF, 5′-ccccatcaagtatttccaagct-3′; 3IR, 5′-gttattgtgttgttgtttttcagtgctta-3′; 3IIF, 5′-aacttccatagctattcattgagtcaaa-3′; 3IIR, 5′- tgagcgatgtggctcggct -3′.

Sequence based-LOH analysis was performed on the pancreatic lesion by direct analysis of the *CDKN1B* mutation.

### Cell culture, transfection, and protein extraction

The human cervical carcinoma HeLa, the rodent p27^KIP1^-negative GH-secreting pituitary adenoma GH3, the human embryonic kidney HEK293 and the human neuroblastoma SH-SY5Y cell lines (American Type Culture Collection, Manassas, VA), were maintained at 37°C in a 5% CO_2_ in complete 10% FCS DMEM.

GH3 (1.5×10^5^ cells/well), HeLa (1.0×10^5^ cells/well), SH-SY5Y (1.30×10^5^ cells/well) and HEK293 (2.5×10^5^ cells/well) cells were plated 24 hours before transfection into 12-well plates. When necessary, 24 hours after seeding cells have been arrested in G1 phase by a 36-hour treatment with either 10 µM (GH3) or 20 µM (HeLa) lovastatin. In all cell lines but HEK293 (see below) transient transfection was performed by Superfect (Qiagen, Milan, Italy). 1.5 µg plasmid and a ratio µg DNA/µl Superfect of 1∶6 following manufacturer's protocol were used. The pRL-TK plasmid (Promega) encoding Renilla luciferase was cotransfected and used for normalization of transfection efficiency. After 3 hours, the medium was changed to DMEM with 2% FCS and incubated for further 24 hours. Cells were then harvested in passive lysis buffer (Promega) and the relative luciferase activity was measured using the Dual-Luciferase Assay System and a GloMax 20/20 luminometer (Promega) according to the manufacturer's instructions.

For expression experiments, HEK293 cells were seeded into 12-well plates, grew to 95% confluence and transfected with Lipofectamine 2000 (Invitrogen, Milan, Italy) following the manufacturer's protocol. Cells were harvested 24 hours post-transfection, lysed in RIPA Buffer supplemented with proteases inhibitors (MgCl_2_ 10 mM, Pepstatin 1 µM, PMSF 1 mM, cOmplete 1X (Roche, Monza, Italy)). Samples were clarified by centrifugation at 13,000 rpm for 5 min at 4°C.

### Western blotting

Concentrations of the HEK293 extracted proteins were determined using the Bio-Rad DC protein assay kit (Bio-Rad Italia, Milan, Italy) following the manufacturer's instructions. For each sample, 20 µg were resuspended in NuPAGE LDS sample buffer and NuPAGE sample reducing agent (Invitrogen), boiled for 10 min at 70°C and resolved by SDS-PAGE on 4–12% NuPAGE gels (Invitrogen) and Mes buffer (Invitrogen). Separated proteins were transferred onto nitrocellulose membrane by Trans-Blot Turbo transfer system (BioRad) that was blocked for 2 hours with 5% non-fat dry milk (BioRad). The membrane was incubated overnight at 4°C with anti-p27^KIP1^ monoclonal antibody (BD Bioscience Heidelberg, Germany) used at 1∶300. Expression was corrected for differences in protein loading by probing blots for 1 hour at RT with mouse anti-ß-actin antibody (clone AC-15 1∶5,000, Sigma-Aldrich, Milan, Italy). Blots were developed using Pierce ECL Substrate (Part No. 32106, Thermo Scientific, Rockford, IL USA) and exposed to CL-XPosure Film (Thermo Scientific).

### RNA isolation from HEK293 cells, reverse transcription, and quantitative real-time PCR (qPCR)

For total RNA extraction, HEK293 cells were resuspended in TRIzol (Invitrogen) and processed according to the manufacturer's instructions. Plasmid DNA contamination was removed by DNase, treating total RNA twice with Turbo DNA free kit (Applied Biosystems, Milan, Italy). One µg of DNase-treated RNA was reverse-transcribed using M-MuLV Reverse Transcriptase RNase H- (F-572S, Finnzymes, Espoo, Finland). qPCR was done with Platinum SYBR Green qPCR SuperMix-UDG (Invitrogen) in an ABI PRISM 7900HT Sequence Detector (Applied Biosystems). A final concentration of 300 nM for both forward and reverse primers was used. Primers for qPCR were qLUCF, 5′-gcctgaagtctctgattaagt-3′; qLUCR, 5′-acacctgcgtcgaaga-3′; qrBActF, 5′-agattactgccctggctcct-3′; qrBActR, 5′-aacgcagctcagtaacagtccg -3′; qhGAPDHF, 5′- ctctctgctcctcctgttcgac-3′; qhGAPDHR, 5′- ctctctgctcctcctgttcgac-3′.

Threshold levels were set at the exponential phase of qPCR using Sequence Detection software, version 2.4 (Applied Biosystems). The amount of each target gene relative to the proper housekeeping gene (HK, rat β-actin or human GAPDH) was determined using a relative standard curve method and the results were expressed as a ratio of target gene/HK. A 38-cycle threshold was set, beyond which the gene was considered undetectable.

### Mutation carrier's RNA, allele specific analysis, and rapid amplification of cDNA ends (5′RACE)

Total RNA from whole blood samples was obtained using Paxgene Blood RNA Kit (Qiagen) following manufacturer protocol, while RNA from paraffin-embedded pancreatic tumor tissue was extracted using a modified RNAzol method, as previously described [43]. RNA was reverse-transcribed as described above. Allele-specific analysis was evaluated by qPCR as described above using two different SYBR assays. A final concentration of 300 nM for both forward primers and 50 mM for the unique reverse primer was used (-456_-453del_wtF 5′- cttcttcgtcagcctccctt-3′; -456_-453del_mutF 5′- cttcttcgtcagcctcccac-3′; -456_-453del-R 5′-agccgctctccaaacctt-3′). Given the different efficiency that may characterize the two different assays, the value of each allele was referred to the genomic DNA expressed as ΔCq (Cq value obtained for mRNA minus Cq value for genomic DNA). The lack of a possible deletion/duplication of the corresponding genomic locus was proven by QMPSFs as described above.

5′RACE was performed using 5′RACE System 2.0 kit (Invitrogen) on whole blood derived total RNA following manufacturer's instructions. Briefly, first strand cDNA was synthesized from 2 µg of mRNA by SuperScriptII RNA polymerase reaction using the specific primers GSP1R (5′- gttaactcttcgtggtcc -3′). After adding an oligo-dC tail to the cDNAs 3′-ends, a PCR reaction has been performed with GSP2R primer (5′-ttctcccgggtctgcacg-3′), coupled with an Abridged Anchor Primer (AAP). The resulting DNA fragments were eluted from agarose gel and analyzed by direct sequencing, as reported above.

### Immunohistochemistry

Immunohistochemistry was performed on an automated immunostainer (Ventana Medical Systems, Frankfurt am Main, Germany), according to the manufacturer's protocols with minor modifications [44] using the monoclonal anti-p27^KIP1^ antibody cited above (1∶1,000). The monoclonal MIB5 antibody (1∶500, Dako, Hamburg, Germany) was used to detect the proliferation antigen Ki-67. Positive controls were used to confirm the adequacy of the staining.

### Cloning and mutagenesis

PCR fragments were obtained by amplification of the mutation carrier with forward and reverse primers containing extra HindIII and NcoI sites, respectively (clonF, 5′- catcataagcttccaccttaaggccgcgct -3′; clonR, 5′- catcatccatggttctcccgggtctgcacg -3′). The PCR product was digested and inserted upstream the luciferase reporter gene into the pGL3 Control Vector (Promega). For expression studies the wild type and mutated 5′UTRs were subcloned into pcDNA3.1/p27HA (kind gift of Prof. Sylvain Meloche, Institute for Research in Immunology and Cancer, Université de Montréal, Canada). The c.-469C>T, c.-428A>T and c.-74insC modifications were introduced by QuikChange II XL kit (Stratagene, La Jolla, CA USA) following manufacturer's protocol.

### Lymphoblastoid cell lines and polysomal RNA extraction

EBV-transformed lymphoblastoid cells were generated by infection of peripheral blood mononuclear cells from the c.-456_-453delCCTT mutation carrier with culture supernatant from the EBV-producing marmoset cell line B95.8 (American Type Culture Collection) and maintained in RPMI 1640 medium (Euroclone, Milano, Italy) supplemented with 10% FBS, 1 mM Na Pyruvate, 10 mM Hepes Buffer, 2 mM Ultraglutamine (Lonza BioWhittaker, Basel, Switzerland), 1% Antibiotic/antimycotic (Gibco, Invitrogen Corporation). Cyclosporin A (CsA, Sandoz Pharmaceuticals AG; Cham, Switzerland) was initially added to the cultures to inhibit T cell growth (final concentration, 0.7 µg/ml).

For polysomal RNA extraction lymphoblastoid cells (25×10^6^) were incubated with 100 µg/ml cycloheximide for 4 minutes, washed once with phosphate buffer saline (PBS), resuspended in lysis buffer [10 mM NaCl, 10 mM MgCl_2_, 10 mM Tris–HCl, pH 7.5, 1% Triton X-100, 1% sodium deoxycholate, 100 µg/ml cycloheximide, 0.2 U/µl RNase inhibitor, 1 mM DTT] and transferred to a microcentrifuge tube. After 5 minutes incubation on ice, the extracts were centrifuged for 10 min at 12,000 g at 4°C. The supernatant was collected and stored at −80°C. The cytoplasmic lysates were fractionated by ultracentrifugation (Sorvall rotor, 100 min at 180,000 g) trough 15–50% linear sucrose gradient containing 30 mM Tris–HCl, pH 7.5, 100 mM NaCl, 10 mM MgCl_2_. Eleven fractions were collected monitoring the absorbance at 254 nm. The RNA in each fraction was isolated after proteinase K treatment, phenol–chloroform extraction and isopropanol precipitation. RNA was resuspended in 30 µl of water. For each fraction 1 µg RNA was reverse-transcribed and analyzed by qPCR using allele-specific assays as reported above.

## Supporting Information

Figure S1 Effect of the *CDKN1B* 5′UTR in HEK293 and SH-SY5Y cell lines. The wild type 5′UTR of the *CDKN1B* gene, but not the c.-456_-453delCCTT containing one, is able to induce luciferase activity in asynchronous SH-SY5Y (A) and HEK293 (B) cell lines. On the right side of each panel the flow cytometry evaluation of the corresponding cell line is shown. Data represent three independent experiments. P-values were calculated using a two-tailed t-test. *p<0,01; EV, empty vector; wt, wild type; error bars, standard deviation.
(PDF)
