# Supplementary figures and images for: A Novel Mutation in the Upstream Open Reading Frame of the CDKN1B Gene Causes a MEN4 Phenotype
Source: PLoS Genet. 2013 Mar 21;9(3):e1003350. doi: 10.1371/journal.pgen.1003350 (PMC3605397; doi:10.1371/journal.pgen.1003350)

A

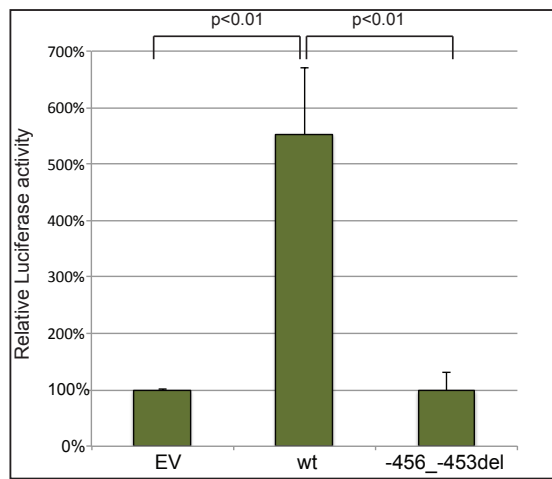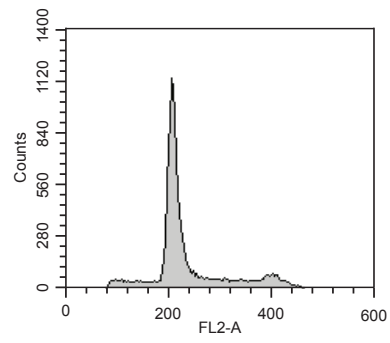

B

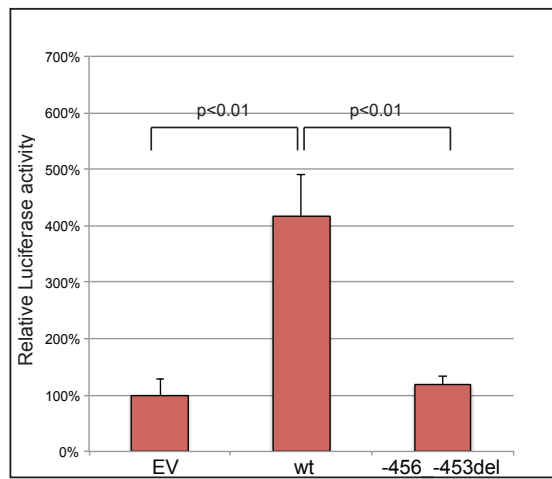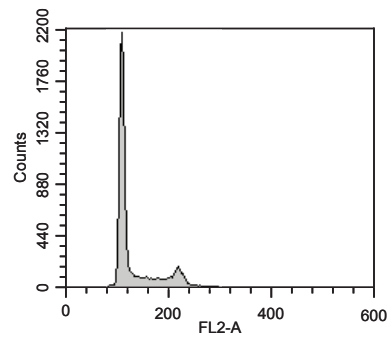

Supplement: Figure S1 — Effect of the CDKN1B 5′UTR in HEK293 and SH-SY5Y cell lines. The wild type 5′UTR of the CDKN1B gene, but not the c.-456_-453delCCTT containing one, is able to induce luciferase activity in asynchronous SH-SY5Y (A) and HEK293 (B) cell lines. On the right side of each panel the flow cytometry evaluation of the corresponding cell line is shown. Data represent three independent experiments. P-values were calculated using a two-tailed t-test. *p<0,01; EV, empty vector; wt, wild type; error bars, standard deviation. (PDF) [file pgen.1003350.s001.pdf]
